# Curcumin attenuates myocardial ischemia–reperfusion injury

**DOI:** 10.18632/oncotarget.23002

**Published:** 2017-12-06

**Authors:** Kun Liu, Honglin Chen, Qing-Sheng You, Qing Ye, Fei Wang, Shuo Wang, Shuang-Long Zhang, Kang-Jun Yu, Qi Lu

**Affiliations:** ^1^ Department of Cardiothoracic Surgery, Affiliated Hospital of Nantong University, Nantong, P.R. China; ^2^ School of Nursing, Nantong University, Nantong, P.R. China; ^3^ Department of Obstetrics and Gynecology, Affiliated Hospital of Nantong University, Nantong, P.R. China; ^4^ Department of Cardiology, Affiliated Hospital of Nantong University, Nantong, P.R. China

**Keywords:** curcumin, myocardial ischemia–reperfusion, inflammation

## Abstract

**Background:**

Cardiovascular diseases (CVDs) are at a badly high-risk of morbidity and mortality in the world.

**Methods:**

Our study was attempted to investigate the cardioprotective role of curcumin. Hearts injury was assessed in isolated hearts and the rats of coronary artery ligated.

**Results and Conclusions:**

The inhibition of pro-inflammatory cytokines was observed by curcumin in coronary artery ligated rats. ST segment was also reduced by curcumin. Triphenyltetrazolium chloride staining (TTC) staining and pathological analysis were also showed that curcumin could dramatically alleviate myocardial injury. Besides, the results *in vitro* also demonstrated that curcumin could improved the function of isolated hearts. Besides, the expressions of inflammation-related pathway in both rats and isolated hearts treated with curcumin were significantly decreased. The present study investigated the protective effects of curcumin on myocardial injury and its mechanism.

## INTRODUCTION

Cardiovascular diseases (CVDs) remain the leading cause of mortality rate around the world. Theoretically, restoring the blood after ischemic myocardium will relieve the symptom of ischemia, however, it may aggravate the damage of myocardial which induced by ischemia/reperfusion (I/R) injury [1]. The myocardial ischemia/reperfusion injury will cause plenty of disease such as functional impairment of hearts, arrhythmia and apoptosis [2]. Inflammation also participate in the process of I/R lesion in isolated hearts [3]. Several research have reported that anti-apoptosis and anti-inflammation action can ameliorate the progress of I/R [4].

As one of the member of the Ras superfamily, the Rho protein drives a several of downstream proteins including Rho-kinase. To our knowledge, RhoA/ROCK pathway controls the activation of NF-κB and MAPK pathways [5, 6]. The RhoA/Rho-kinase pathway was involved in many physiological function including hypertension, heart failure and myocardial hypertrophy [7]. Curcumin, extracted from the rhizome of the Curcuma longa Linnéa, is one of the polyphenolic flavonoid which mainly constituent by turmeric. Curcumin has plenty of biological functions, such as antioxidant, anti-inflammatory, anti-tumorigenic activities [8, 9]. However, there has been no research to explore whether curcumin is beneficial for ischemia/reperfusion. Therefore, our study was to evaluated the effect of curcumin on I/R-induced heart injury and investigated its potential mechanism.

## RESULTS

### Effect of curcumin on myocardial infarct size

**Figure 1:**
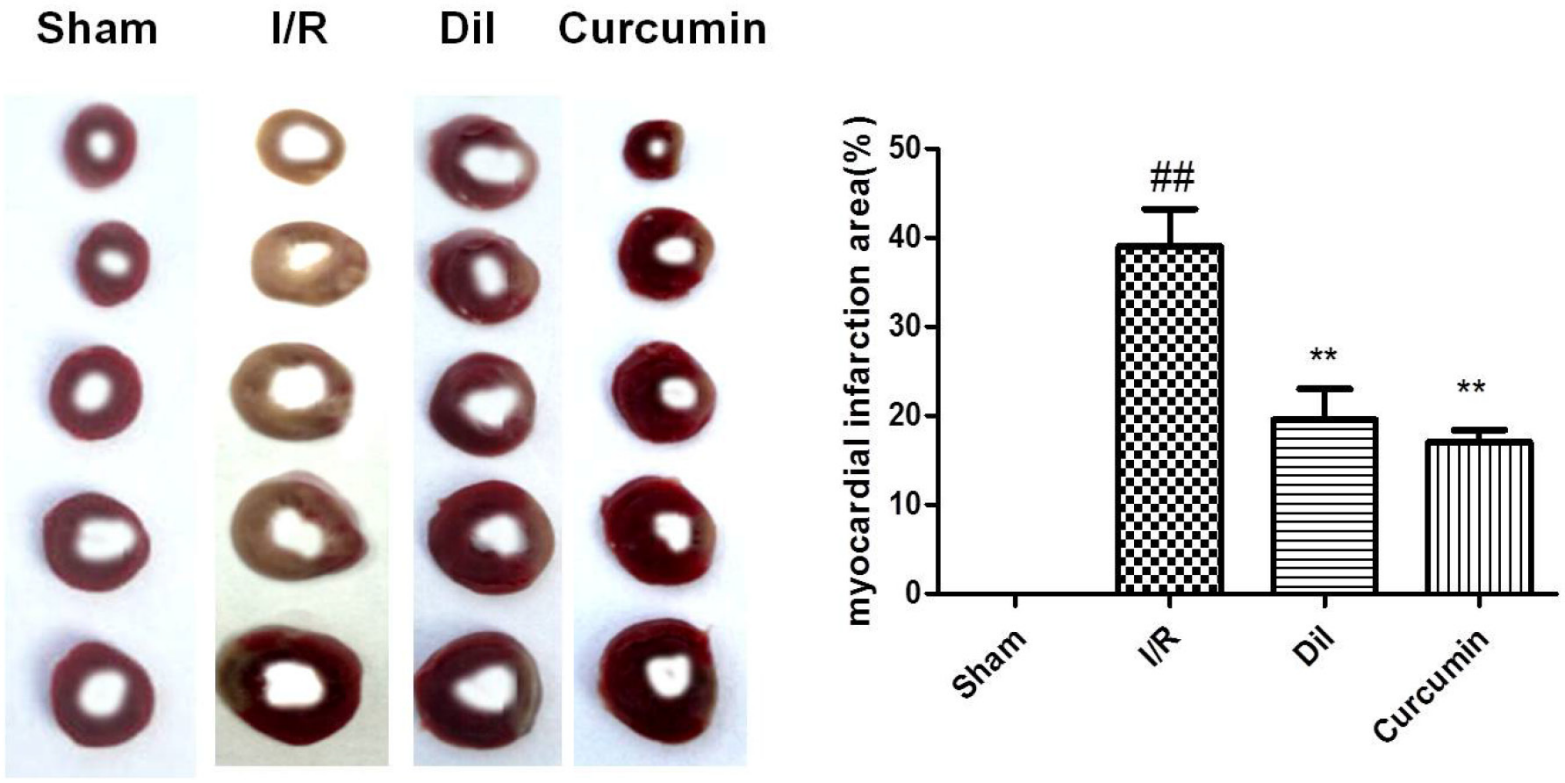

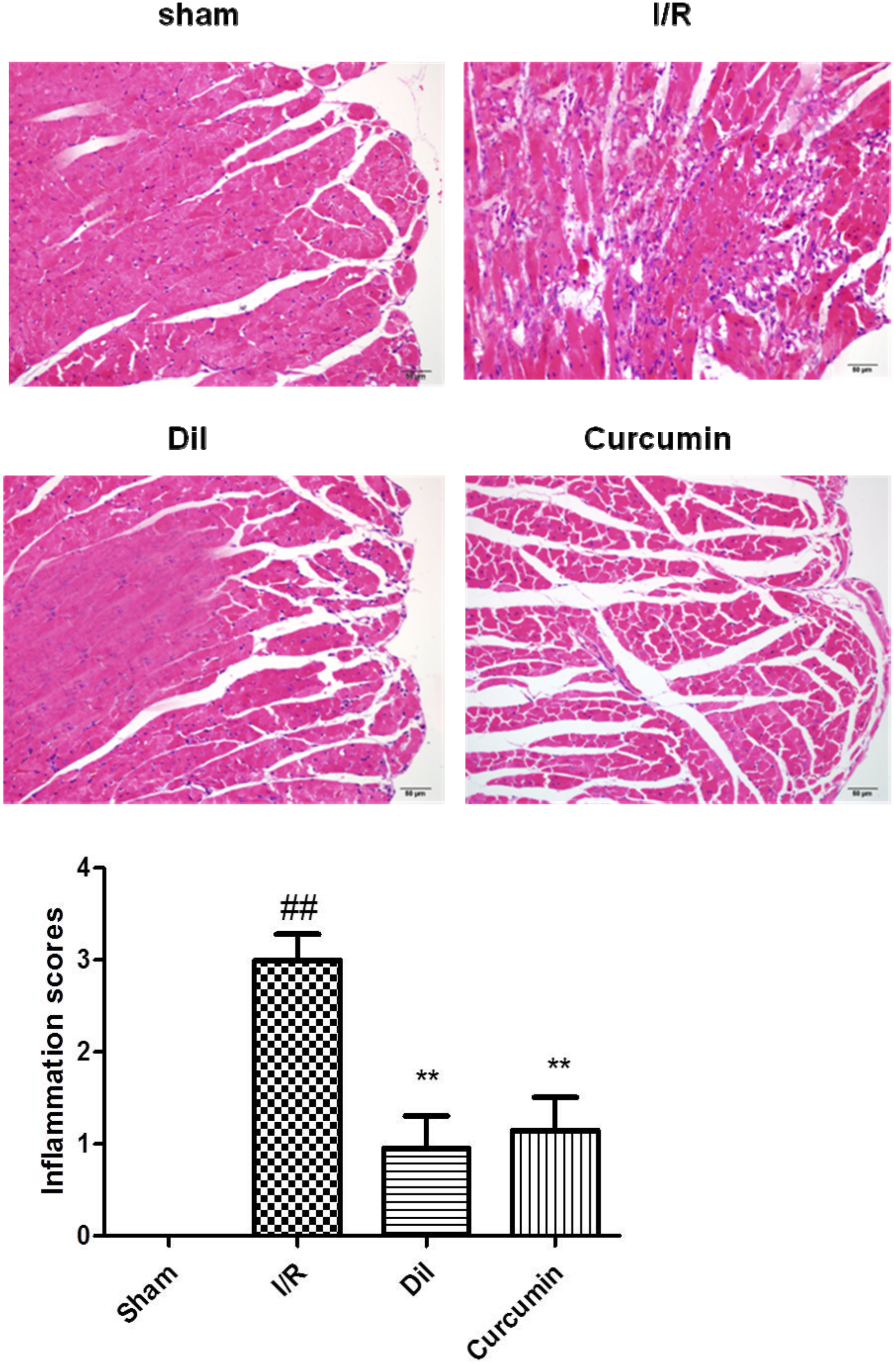

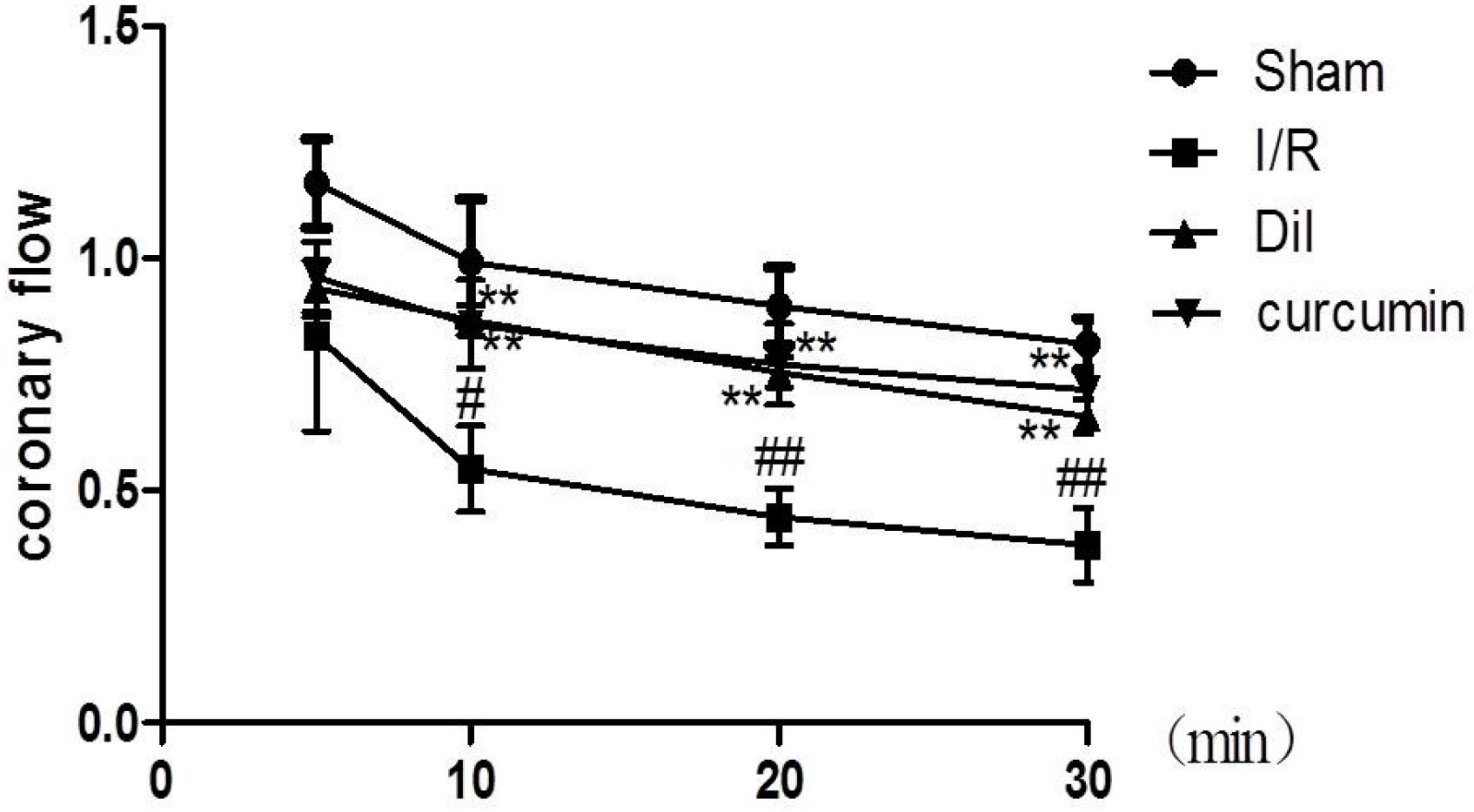

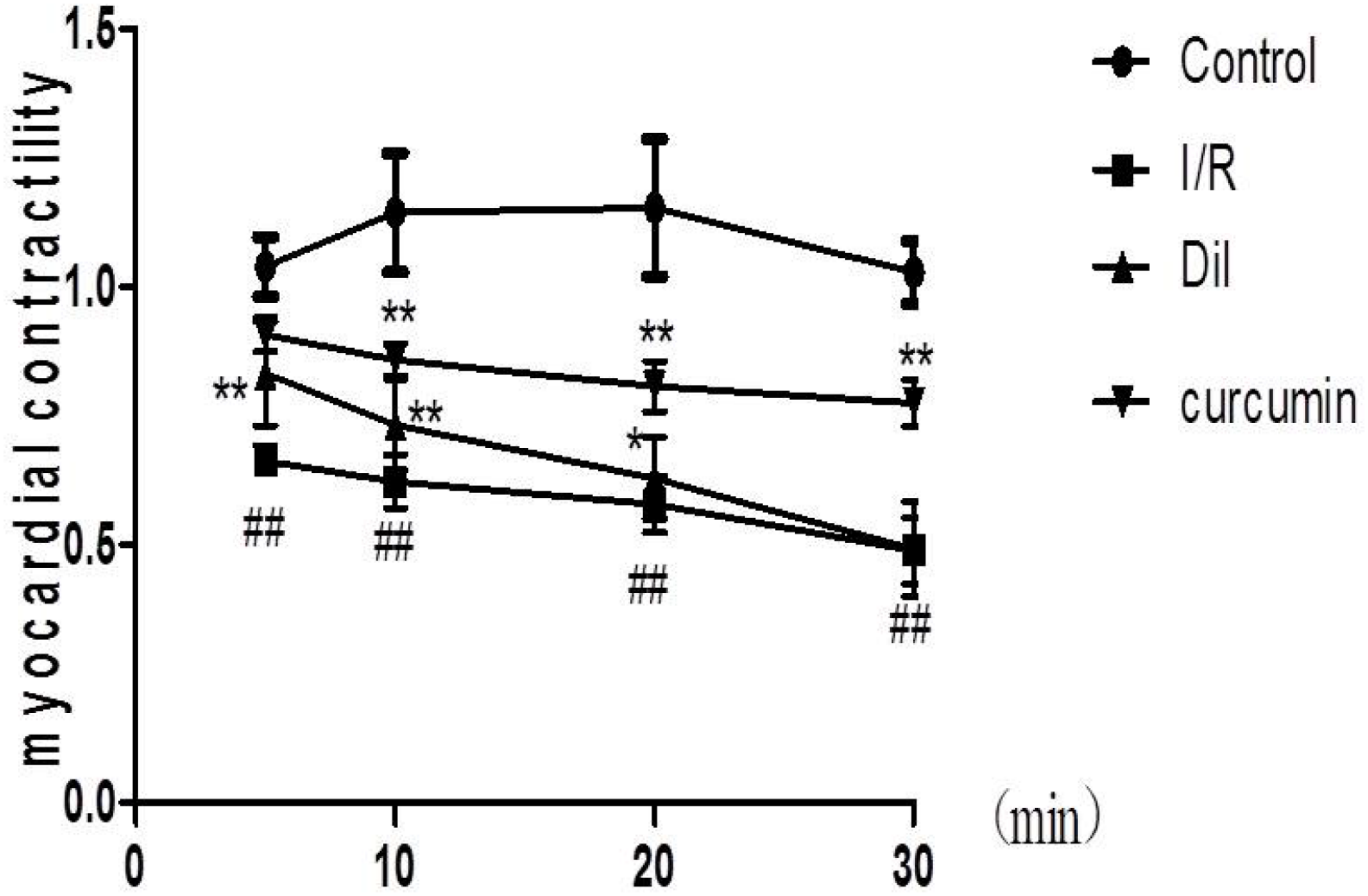
**(A)** Effect of curcumin on myocardial infarct size in I/R-induced isolated heart. The data are expressed as mean values ± SDs. ^#^p < 0.05, ^##^p < 0.01 compared with control group. ^*^p < 0.05, ^**^p < 0.01 compared with I/R group. **(B)** Effect of curcumin on myocardial histology. Original magnification (×200). The data are expressed as mean values ± SDs. ^#^p < 0.05, ^##^p < 0.01 compared with control group. ^*^p < 0.05, ^**^p < 0.01 compared with I/R group. **(C)** Effect of curcumin on coronary flow in I/R-induced isolated heart. The data are expressed as mean values ± SDs. ^#^p < 0.05, ^##^p < 0.01 compared with control group. ^*^p < 0.05, ^**^p < 0.01 compared with I/R group. **(D)** Effects of curcumin on myocardial contractility in I/R-induced isolated heart. The data are expressed as mean values ± SDs. ^#^p < 0.05, ^##^p < 0.01 compared with control group. ^*^p < 0.05, ^**^p < 0.01 compared with I/R group.

TTC staining was an important index for the development of ischemic injury. As it shown in Figure 1A, there was scarce percent of infarct in the sham group, whereas the infarct size in I/R group was increased than that in control group. On the contrary, treatment with Dil and curcumin notably reduced the infarct size.

### Effect of curcumin on myocardial histology

As illustrated in Figure 1B, the tissues in sham group showed the integrated myocardial membrane, a well-balanced myofibrillar structure and a continuous adjacent myofibrils. However, the hearts which suffered from ligation exhibited plenty of inflammatory cells, myocardial cell swelling or degeneration, cardiac necrosis and loss of transverse striations. Curcumin and Dil significantly ameliorate the pathological changes above.

### Effect of curcumin on coronary flow in I/R-induced isolated heart

Myocardial ischemia followed reperfusion in isolated heart caused dramatically decrease in coronary flow rate noted. The alteration caused by curcumin were illustrated in Figure 1C, the coronary flow in I/R group was dramatically decreased (P<0.01), which was slightly less than that in 10 min (P<0.05). By contrast, administration of Dil and curcumin significantly increased coronary flow.

### Effects of curcumin on myocardial contractility in I/R-induced isolated heart

Myocardial contractility of isolated hearts was depicted in Figure 1D. As compared with control group, the blockade of K-H flow markedly limited the contractility of isolated hearts in I/R group. Nevertheless, the intervention with curcumin dramatically elevated myocardial contractility.

### Effects of curcumin on ST-segment elevation

**Figure 2:**
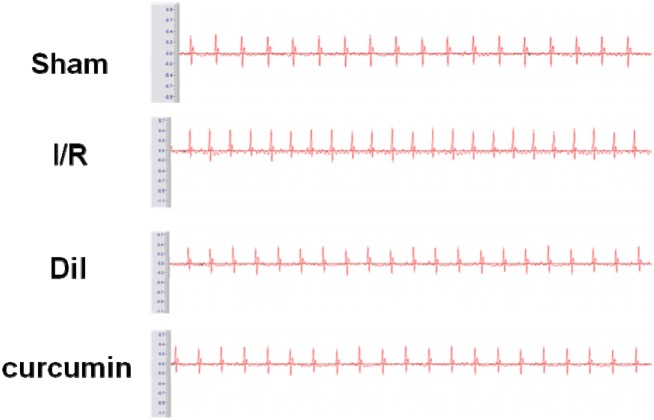
Effects of curcumin on ST-segment elevation

The result of electrocardiographic was revealed in Figure 2. The ST-segment was elevated and R-amplitude was decreased in I/R group compared with those in the control group. Curcumin obviously ameliorated the above phenomenon, which partially evidenced the protective effects.

### Effects of curcumin on inflammatory cytokines in serum

**Figure 3:**
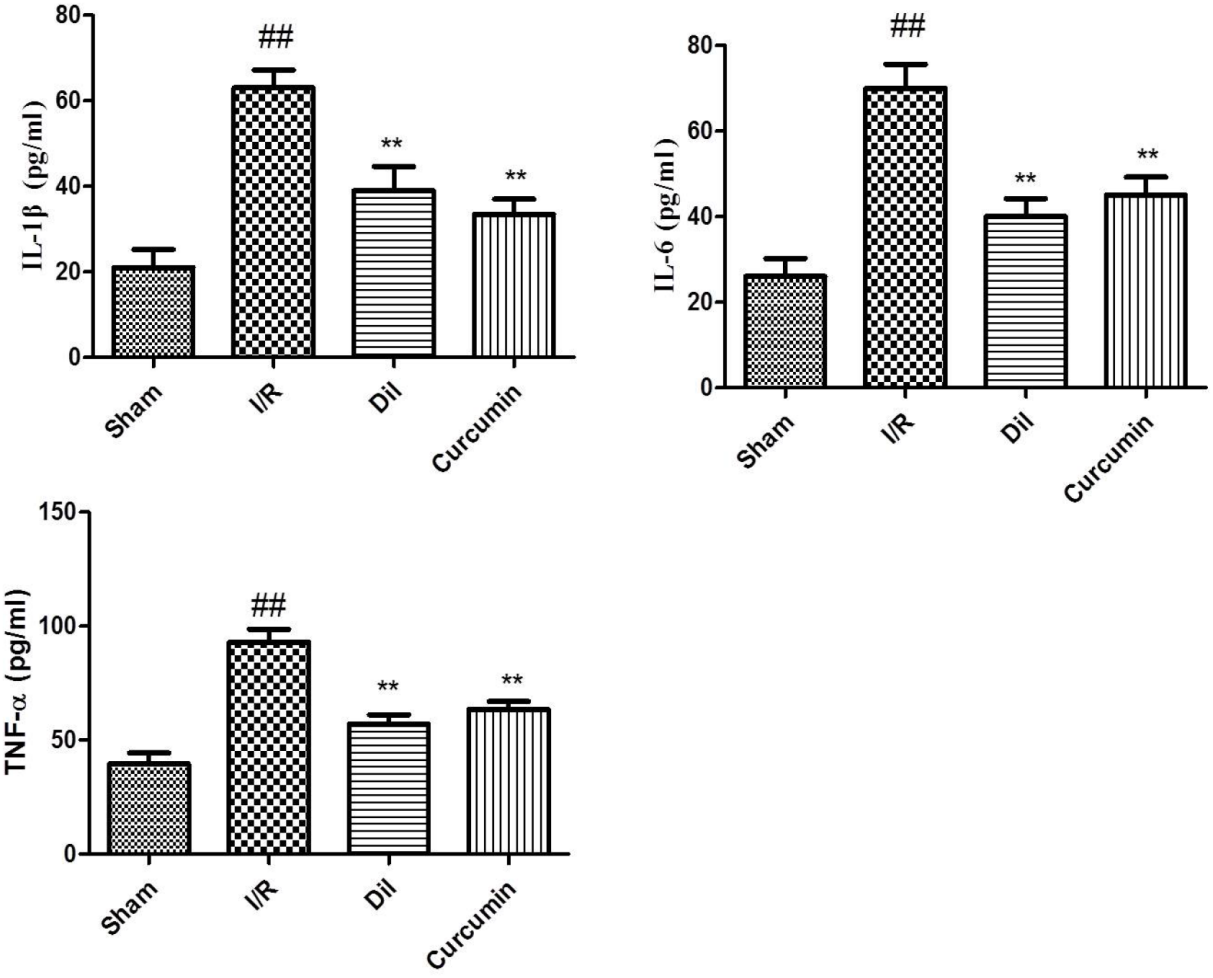

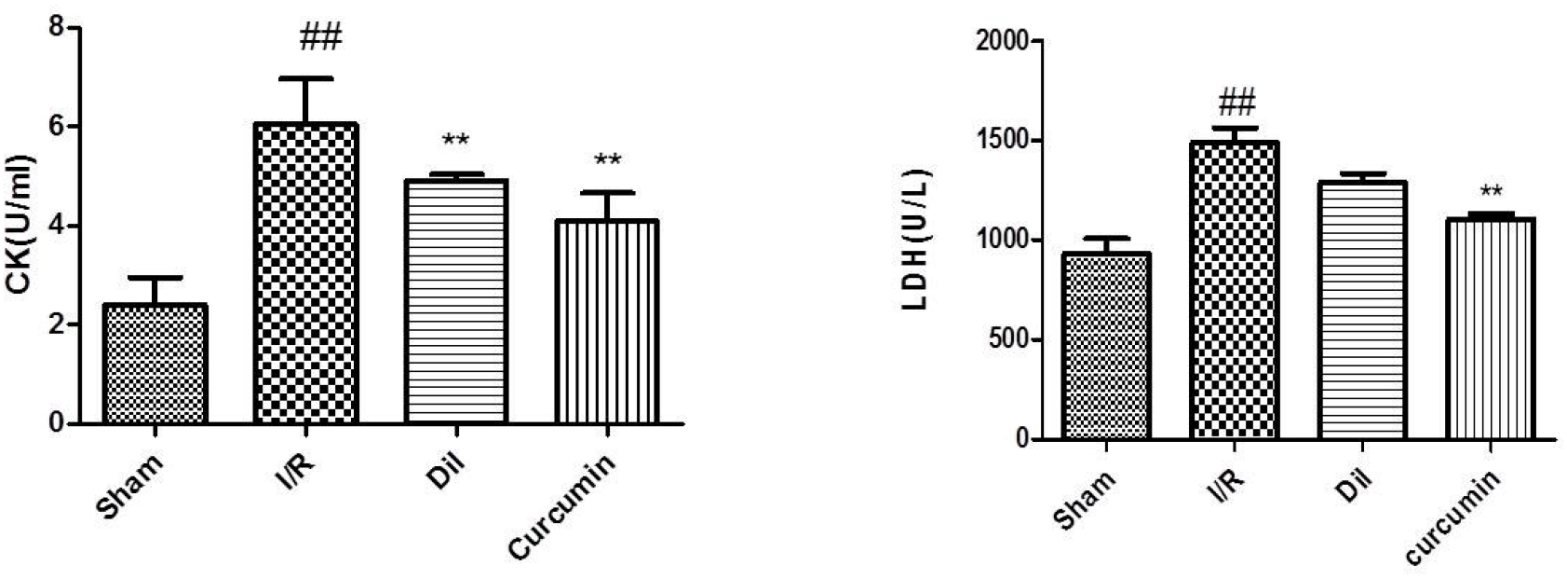
**(A)** Effects of curcumin on inflammatory cytokines in serum. The data are expressed as mean values ± SDs. ^#^p < 0.05, ^##^p < 0.01 compared with control group. ^*^p < 0.05, ^**^p < 0.01 compared with I/R group. **(B)** Effects of curcumin on cardiac marker enzymes. The data are expressed as mean values ± SDs. ^#^p < 0.05, ^##^p < 0.01 compared with control group. ^*^p < 0.05, ^**^p < 0.01 compared with I/R group.

Inflammatory cytokines are one of the major index in myocardial animal model. To determine whether curcumin could inhibit inflammatory responses during myocardial I/R injury, levels of TNF-α, IL-6 and IL-1β in serum were assessed. It was proved that there were significant increases of the serum levels of TNF-α, IL-6 and IL-1β in I/R group. To the respective, the serum levels of TNF-α, IL-6 and IL-1β were effectively decreased in curcumin-treated group compared with those in I/R group rats. Our results indicated that curcumin reduced the inflammatory cytokine in I/R injury rats (Figure 3A).

### Effects of curcumin on cardiac marker enzymes

The levels of CK and LDH in serum were measured in order to detect myocardial injury. As depicted in Figure 3B, the activities of CK and LDH were obviously increased in I/R injury compared with those in sham group. As expected, curcumin significantly decreased the levels of CK and LDH compared with I/R group.

### Effects of curcumin on RhoA/Rho kinase pathway in I/R-induced heart

**Figure 4:**
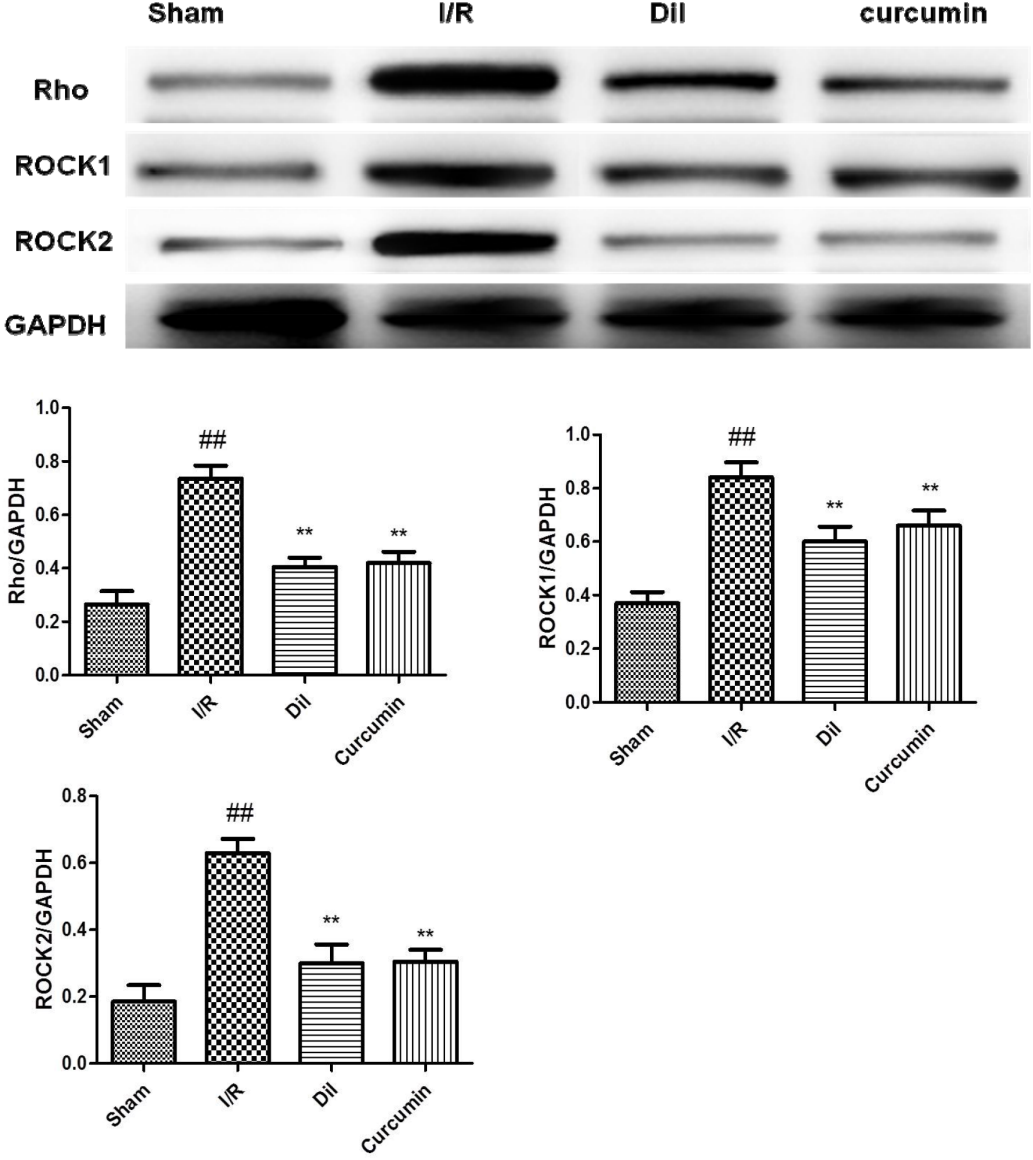

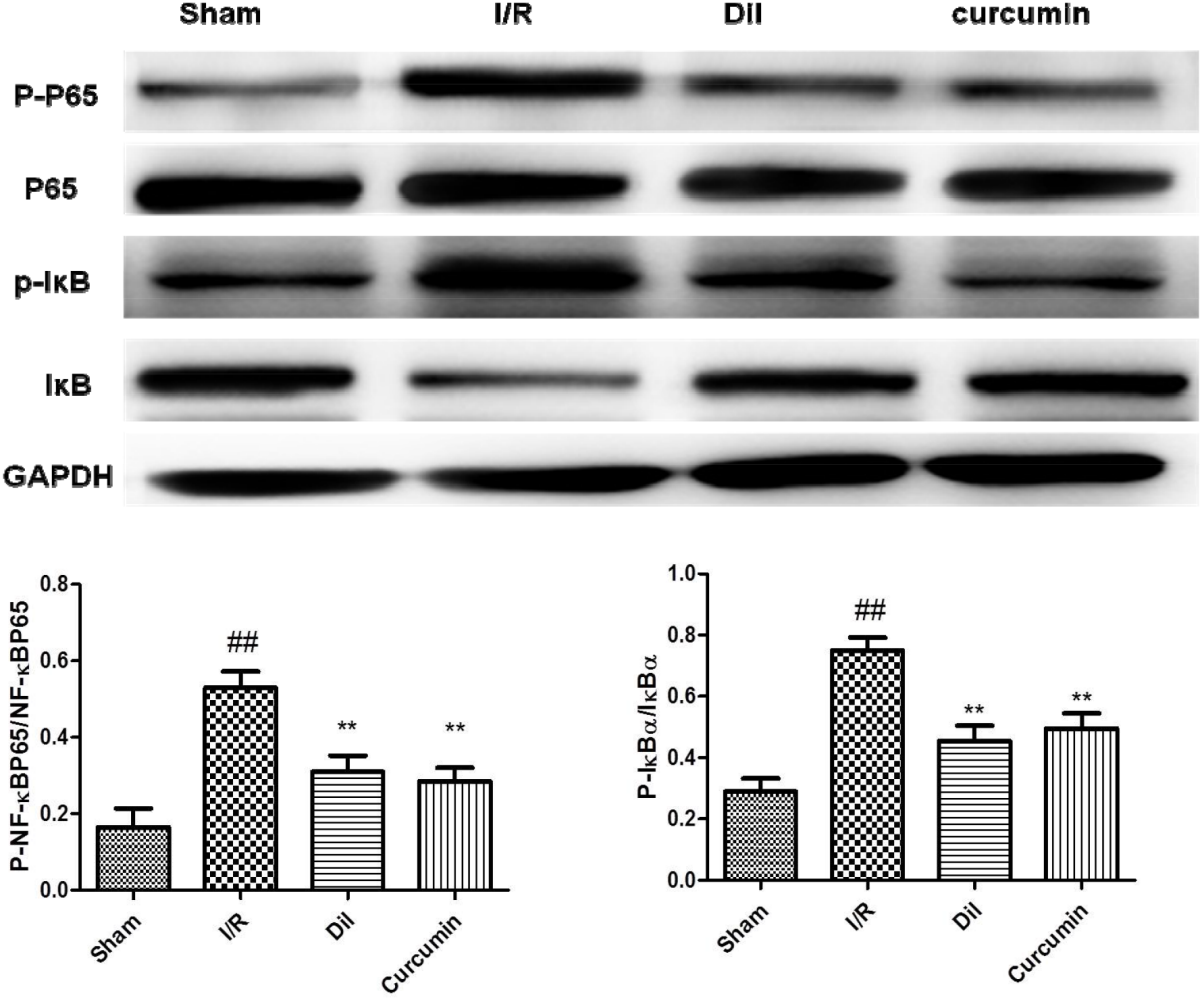
**(A)** Effects of curcumin on RhoA/Rho Kinase Pathway in I/R-induced heart *in vivo* **(a)** *in vitro* **(b).** The data are expressed as mean values ± SDs. ^#^p < 0.05, ^##^p < 0.01 compared with control group. ^*^p < 0.05, ^**^p < 0.01 compared with I/R group. **(B)** Effects of curcumin on NF-κB activation in I/R-induced heart *in vivo* **(a)** *in vitro* **(b).** The data are expressed as mean values ± SDs. ^#^p < 0.05, ^##^p < 0.01 compared with control group. ^*^p < 0.05, ^**^p < 0.01 compared with I/R group.

RhoA/Rho kinase pathway participates in the mediation of inflammatory cascade. We found that the content of RhoA, ROCK1 and ROCK2 were significantly increased in heart tissues after I/R stimulation. However, treatment with Dil and curcumin obviously ameliorated these situations. The obtained results demonstrated that curcumin successfully blocked the expressions of RhoA, ROCK1 and ROCK2. (Figure 4A).

### Effects of curcumin on NF-κB activation in I/R-induced heart

To elucidate the downstream mechanism of curcumin on I/R-induced heart in rats, the phosphorylations of IκB and NF-κB were detected. The protein levels of p-IκB and p-NF-κB and were upregualted in I/R group compared with those in control group. While curcumin significantly exerted a suppressive effect on I/R-induced phosphorylated IκB and NF-κB promotions. Simultaneously, Dil administrations were demonstrated to have inhibited effects on the phosphorylations of IκB and NF-κB (Figure 4B).

## DISCUSSION

Currently, there has been various intervetions to cardiovascular diseases including thrombolytic therapy and coronary artery bypass grafting. However, the curative effect of the I/R injuries with these treatments is not good. Thus, the treatment of I/R injury is needed further research.

Under physiological conditions, coronary flow and cardiac contractility are the reliable indices to reflect the basal function. Additionally, they are also related to myocardial oxygen consumption and energy metabolism. Isolated hearts without the perfusion of oxygen-containing K-H solution were desperate for nutrients and oxygen, which contributes to the lower coronary flow and myocardial contractility. While the blockade of ROCK effectively attenuated the coronary flow and cardiac contractility in I/R injury, which was in accordance with cardioprotective effects of isolated rat hearts observed in our previous investigation [10]. The results of the TTC staining and ST-segment elevation of the present work also confirmed the cardioprotective effect of curcumin.

Rho-kinase (ROCK), a serine/threonine protein kinase, includes two isoforms ROCK1 and ROCK2. ROCK, a member of the Rho family of small G-proteins, is activated by RhoA [11]. It is widely acknowledged that ROCK expression can be blocked by the competitive inhibitor Y-27632. Former literatures demonstrated that the treatment with ROCK inhibitors prolongs cardiac allograft survival [12]. As the downstream molecule of ROCK, NF-κB plays an essential role in the regulation of inflammatory and apoptotic progression in myocardial ischemia [13, 14]. The activation of NF-κB is triggered by the phosphorylation and degradation of the IκBα [15]. NF-κB governs the mediation of inflammatory cytokine production, oxidative stress and apoptotic reaction [16]. Evidence has suggested that Rho/ROCK/NF-κB pathway was involved in cardiomyocytes and in experimental myocardial infarction [17]. Our results confirmed that curcumin successfully blocked the expression of ROCK and the inhibition of ROCK might conduce to the suppression of NF-κB activation.

In conclusion, the present study successfully to investigate the cardioprotective role of curcumin on I/R injury-induced isolated heart tissues and tried to elucidate the potential mechanism of ROCK/NF-κB induced apoptotic pathogenesis. Further research are warranted for more details in the future.

## MATERIALS AND METHODS

### Reagents

Curcumin was obtained from Sigma-Aldrich (Saint Louis, MO, USA). All the antibodies were provided by Cell Signaling Technology (Danvers, USA).

### Animals

Sprague-Dawley rats(250-300 g) were purchased from Laboratory animal center, Nantong university (Nantong, China) were fed in 23 ± 2°C with a 12-h light/dark cycle. Water and food were provided *ad libitum*. All the experimental procedures were performed according to the National Institutes of Health Guidelines for the Care and Use of Laboratory Animals.

Ethical standards statement: All animal operations were performed according to Nantong university (NJU.2016-003).

### Experimental protocol of the left coronary artery ligation

Rats were anesthetized and then restrained on the fixator. Incised the trachea and placed a catgut for further use. Then, open the skin above the heart, and blunt-dissection the ribs under the skin. Meanwhile, tie a line on the left anterior descending coronary. After that, a positive pressure respirator was given to the experimental rats. When it become conscious of a regular heart rhythm, the thoracic cavity was sutured. The sham group of the mice were not tied. The mice were divided into 4 groups as follows: sham group, model group (I/R), Dil group (I/R + Dil 10 mg/kg), curcumin group (I/R + curcumin 300 mg/kg). Curcumin and Dil were given for 5 days after surgery.

24 h after the operation, the blood samples were collected and centrifuged at 3500 g for 15 min, and the supernatant were stored at −80°C for further analysis. Then collected the hearts of the mice for other analysis.

### Experimental protocol of langendorff-perfused

Sprague Dawley rats (250-300 g) were anesthetized by chloral hydrate (350 mg/kg) and restrained. 250 U/kg of heparin were given intraperitoneally in order to prevent coagulation of the blood. The heart was then excised quickly and hung it on the Langendorff’s apparatus. The hearts were retrogradely perfused under a constant perfusion pressure 70 cm H_2_O with complex Krebs-Henseleit(K-H) buffer which consisted of: 118 mM NaCl, 1.2 mM KH_2_PO_4_, 4.7 mM KCl, 1.7 mM CaCl_2_, 1.2 mM MgSO_4_, 20 mM sodium acetate and 10 mM glucose with 95 % O_2_ plus 5 % CO_2_ at 37°C (pH 7.4). Heart and perfusate were kept in 37°C adopt a water jacket. Thereafter, the isolated hearts were perfused by K-H solution with the pressure of 80–90 mm Hg.

The hearts were randomly assigned into four groups: control group, I/R group, I/R + Dil group, I/R+ curcumin group. Hearts in control group were perfused for the 80 min stabilization period. Hearts in I/R group were allowed to equilibrate for 20 min, and then the global ischemia was performed for 30 min. Consequently, reperfusion was conducted for 30 min using K-H buffer. I/R + Dil group or I/R+ curcumin group were treated in accordance with the procedures in I/R group, whereas reperfusion was subjected to Dil (0.2 mg/kg) or curcumin (0.5 mg/kg) dissolved K-H buffer for 30 min.

### Detection of coronary flow and myocardial contractility

The coronary flow (CF) rate was observed at 5 min, 10 min, 20 min and 30 min after reperfusion by measuring the effluent which flowed from the right atrium into a measuring cylinder. The heart with the rate of > 8 ml/min during the stabilization period was selected. Coronary effluent (CE) was harvested at end of pending test. Moreover, frog heart clip was applied to record the contractility of myocardial during the experiment period.

### Electrocardiogram (ECG) measurement

ECG was detected by BL-420S Biologic Function Experiment system (Chengdu, China). ST Segment elevation was seemed as the symbol of ischemia which was recorded by ECG.

### Measurements of cardiac output

Rats were anaesthetized and placed on a heating pad. The ascending aorta was dissected and a transonic perivascular MA2.5 PSL flow probe (Transonic Systems, USA) was put beside the aorta. The ultrasound transmission gel was given into the side of the probe and the aorta. The data was collected by TS420 flowmeter and PowerLab recording unit.

### Determination of myocardial infarct size

The cardiac infarct area was evaluated using TTC (Sigma, St. Louis, MO, USA) staining. Generally, The heart was put in -20°C for 15 min and chopped into 5 pieces parallel to the coronary sulcus. All slices were incubated with PBS with 2 % TTC at 37°C for 15 min in the dark atmosphere. TTC stained and non-TTC stained area were analyzed by an Image-Pro Plus image analysis software (Version 4.1, Media Cybernetics, LP, USA). The ratio of infarcted myocardium to risk region was calculated as the whole myocardial tissues (infarct area/whole heart area) × percentage.

### Determination of inflammatory cytokines in serum

The levels of IL-6, IL-1β and TNF-α in serum were measured by ELISA kits according to the manufacturer’s instructions. The concentrations of the cytokines were quantified by standard curves. The absorbancy was detected at 450 nm.

### Determination of cardiac marker enzymes

CK and LDH activities in serum were assayed by commercial kits according to the manufacturer’s instructions.

### Histological examination of myocardium

The hearts were harvest and fixed in 10% (V/V) neutral buffered formalin solution. It was embedded with paraffin and cut into 4 μm thicknesses. The tissues were stained with H&E and observed by light microscopy (Nikon, Tokyo, Japan). The scoring inflammatory cell infiltration was as follows: 0, no cells; 1, a few cells; 2, a ring of cells one cell layer deep; 3, a ring of cells two to four cell layers deep; and 4, a ring of cells more than four cell layers deep.

### Western blot

The heart was homogenized and lysed in a RIPA buffer (Beyotime, Nanjing, China). Then, the tissues was centrifugation at 12 000 rpm for 20 min and collected the supernatant. The concentration of the protein were determined by a BCA protein assay (Beyotime, Nanjing, China). The samples were separated by 10% sodium dodecyl sulphate polyacrylamide gels and then transferred onto nitrocellulose membranes. The membranes were blocked with 5% skim milk in for 2 h and incubated with specific antibodies at 4°C for a whole night. After washing the membranes for 4 times on the second day, the blots were incubated with horseradish peroxidase-conjugated second antibodies for 2 h. The content of the protein was showed by a gel imaging system (Tanon Science & Technology Co., Ltd., China).

### Statistical analysis

Data are expressed as means ± SDs of at least three separate experiments. Statistical comparisons between experimental groups were performed by ANOVA with Tukey multiple comparison test. A value of P < 0.05 was considered statistically significant.

## References

[1] Hu Q, Wei B, Wei L, Hua K, Yu X, Li H, Ji H (2015). Sodium tanshinone IIA sulfonate ameliorates ischemia-induced myocardial inflammation and lipid accumulation in Beagle dogs through NLRP3 inflammasome. Int J Cardiol.

[2] Zhu L, Wei T, Gao J, Chang X, He H, Luo F, Zhou R, Ma C, Liu Y, Yan T (2015). The cardioprotective effect of salidroside against myocardial ischemia reperfusion injury in rats by inhibiting apoptosis and inflammation. Apoptosis.

[3] Weinreuter M, Kreusser MM, Beckendorf J, Schreiter FC, Leuschner F, Lehmann LH, Hofmann KP, Rostosky JS, Diemert N, Xu C, Volz HC, Jungmann A, Nickel A (2014). CaM Kinase II mediates maladaptive post-infarct remodeling and pro-inflammatory chemoattractant signaling but not acute myocardial ischemia/reperfusion injury. EMBO Mol Med.

[4] Hua K, Sheng X, Li TT, Wang LN, Zhang YH, Huang ZJ, Ji H (2015). The edaravone and 3-n-butylphthalide ring-opening derivative 10b effectively attenuates cerebral ischemia injury in rats. Acta Pharmacol Sin.

[5] Chen T, Wang R, Jiang W, Wang H, Xu A, Lu G, Ren Y, Xu Y, Song Y, Yong S, Ji H, Ma Z (2016). Protective effect of astragaloside IV against paraquat-induced lung injury in mice by suppressing Rho signaling. Inflammation.

[6] Fierro C, Novoa U, Gonzalez V, Ocaranza MP, Jalil JE (2016). Simultaneous Rho kinase inhibition in circulating leukocytes and in cardiovascular tissue in rats with high angiotensin converting enzyme levels. Int J Cardiol.

[7] Lin G, Craig GP, Zhang L, Yuen VG, Allard M, McNeill JH, MacLeod KM (2007). Acute inhibition of Rho-kinase improves cardiac contractile function in streptozotocin-diabetic rats. Cardiovasc Res.

[8] Wang Y, Lu Z, Wu H, Lv F (2009). Study on the antibiotic activity of microcapsule curcumin against foodborne pathogens. Int J Food Microbiol.

[9] Zhao S, Ma L, Cao C, Yu Q, Chen L, Liu J (2017). Curcuminloaded redox response of self-assembled micelles for enhanced antitumor and anti-inflammation efficacy. Int J Nanomedicine.

[10] Chang X, Zhang K, Zhou R, Luo F, Zhu L, Gao J, He H, Wei T, Yan T, Ma C (2016). Cardioprotective effects of salidroside on myocardial ischemia-reperfusion injury in coronary artery occlusion-induced rats and Langendorff-perfused rat hearts. Int J Cardiol.

[11] Chen T, Guo Q, Wang H, Zhang H, Wang C, Zhang P, Meng S, Li Y, Ji H, Yan T (2015). Effects of esculetin on lipopolysaccharide (LPS)-induced acute lung injury via regulation of RhoA/Rho Kinase/NF-small ka, CyrillicB pathways *in vivo* and *in vitro*. Free Radic Res.

[12] Ohki S, Iizuka K, Ishikawa S, Kano M, Dobashi K, Yoshii A, Shimizu Y, Mori M, Morishita Y (2001). A highly selective inhibitor of Rho-associated coiled-coil forming protein kinase, Y-27632, prolongs cardiac allograft survival of the BALB/c-to-C3H/He mouse model. J Heart Lung Transplant.

[13] Deng XY, Chen JJ, Li HY, Ma ZQ, Ma SP, Fu Q (2015). Cardioprotective effects of timosaponin B II from Anemarrhenae asphodeloides Bge on isoproterenol-induced myocardial infarction in rats. Chem Biol Interact.

[14] Zhu L, Chen T, Chang X, Zhou R, Luo F, Liu J, Zhang K, Wang Y, Yang Y, Long H, Liu Y, Yan T, Ma C (2016). Salidroside ameliorates arthritis-induced brain cognition deficits by regulating Rho/ROCK/NF-kappaB pathway. Neuropharmacology.

[15] Jiang Q, Yi M, Guo Q, Wang C, Wang H, Meng S, Liu C, Fu Y, Ji H, Chen T (2015). Protective effects of polydatin on lipopolysaccharide-induced acute lung injury through TLR4-MyD88-NF-kappaB pathway. Int Immunopharmacol.

[16] Zhang K, Liu J, You X, Kong P, Song Y, Cao L, Yang S, Wang W, Fu Q, Ma Z (2016). P2X7 as a new target for chrysophanol to treat lipopolysaccharide-induced depression in mice. Neurosci Lett.

[17] Chorianopoulos E, Heger T, Lutz M, Frank D, Bea F, Katus HA, Frey N (2010). FGF-inducible 14-kDa protein (Fn14) is regulated via the RhoA/ROCK kinase pathway in cardiomyocytes and mediates nuclear factor-kappaB activation by TWEAK. Basic Res Cardiol.

